# Lithium alters brain activation in bipolar disorder in a task- and state-dependent manner: an fMRI study

**DOI:** 10.1186/1744-859X-4-14

**Published:** 2005-07-19

**Authors:** Peter H Silverstone, Emily C Bell, Morgan C Willson, Sanjay Dave, Alan H Wilman

**Affiliations:** 1 Department of Psychiatry, Faculty of Medicine, University of Alberta, 1E1.07 MacKenzie Center, 8440-112 Street, Edmonton, Alberta, T6G 2B7, Canada; 2 Department of Biomedical Engineering, Faculty of Medicine, University of Alberta, 1071 Research Transition Facility, 8308-114 Street, Edmonton, Alberta, T6G 2V2, Canada

**Keywords:** bipolar disorder, euthymic, depression, functional magnetic resonance imaging, BOLD.

## Abstract

**Background:**

It is unknown if medications used to treat bipolar disorder have effects on brain activation, and whether or not any such changes are mood-independent.

**Methods:**

Patients with bipolar disorder who were depressed (n = 5) or euthymic (n = 5) were examined using fMRI before, and 14 days after, being started on lithium (as monotherapy in 6 of these patients). Patients were examined using a word generation task and verbal memory task, both of which have been shown to be sensitive to change in previous fMRI studies. Differences in blood oxygenated level dependent (BOLD) magnitude between the pre- and post-lithium results were determined in previously defined regions of interest. Severity of mood was determined by the Hamilton Depression Scale for Depression (HAM-D) and the Young mania rating scale (YMRS).

**Results:**

The mean HAM-D score at baseline in the depressed group was 15.4 ± 0.7, and after 2 weeks of lithium it was 11.0 ± 2.6. In the euthymic group it was 7.6 ± 1.4 and 3.2 ± 1.3 respectively. At baseline mean BOLD signal magnitude in the regions of interest for the euthymic and depressed patients were similar in both the word generation task (1.56 ± 0.10 and 1.49 ± 0.10 respectively) and working memory task (1.02 ± 0.04 and 1.12 ± 0.06 respectively). However, after lithium the mean BOLD signal decreased significantly in the euthymic group in the word generation task only (1.56 ± 0.10 to 1.00 ± 0.07, p < 0.001). Post-hoc analysis showed that these differences were statistically significant in Broca's area, the left pre-central gyrus, and the supplemental motor area.

**Conclusion:**

This is the first study to examine the effects of lithium on brain activation in bipolar patients. The results suggest that lithium has an effect on euthymic patients very similar to that seen in healthy volunteers. The same effects are not seen in depressed bipolar patients, although it is uncertain if this lack of change is linked to the lack of major improvements in mood in this group of patients. In conclusion, this study suggests that lithium may have effects on brain activation that are task- and state-dependent. Given the small study size and the mildness of the patient's depression these results require replication.

## Background

Studies suggest that there are trait abnormalities in cognitive functioning in bipolar disorder, i.e. cognitive changes which are seen when patients are either manic or depressed but which are also seen when patients are euthymic. These are seen in studies of sustained attention, executive functioning, and verbal learning [1,2]. The evidence to date suggests that there are trait cognitive abnormalities in bipolar patients, although it is possible these can be exacerbated by medication and the duration of illness [3]. However, it should be recognized that some of the treatments for bipolar disorder, particularly lithium, can also cause cognitive deficits particularly in tests of verbal memory [4,5].

It remains uncertain how these cognitive deficits may be reflected in functional changes in brain activation. One study comparing depressed and euthymic bipolar patients found regional cerebral blood flow (rCBF) changes in both groups in multiple brain regions [6]. Another study utilizing fMRI examined patients who were depressed, manic, and euthymic and suggested there was a trait abnormality in the prefrontal cortex [7]. An fMRI study comparing euthymic patients to controls also suggested changes in limbic and visual cortex may represent trait abnormalities of brain functioning [8]. Another fMRI study in euthymic bipolar patients found significantly increased activation compared to controls in a working memory task in several different brain regions [9]. Thus, studies to date support suggestions of underlying functional changes in bipolar patients that to some extent may reflect the well recognized cognitive deficits.

It is of considerable interest to determine whether functional changes seen in bipolar patients may be attenuated by treatment, but to date there have been no studies examining the possible effects of treatment upon brain activation. Lithium remains one of the primary treatments for bipolar disorder [10], and as noted also can have independent cognitive effects. In a previous study we have shown that in healthy volunteers treatment with lithium for 2 weeks led to a decrease in mean BOLD magnitude in a word generation task (unpublished information). However, one of the problems of examining patients before and after treatment is that if the mood changes following medication, it can be difficult to know what the effects of medication alone are. For these reasons the present study examines both bipolar patients who are depressed and those who are euthymic. Given previous findings about cognitive deficiencies, the two cognitive tests used in the present study were firstly one that focused on sustained attention (working memory task) and the second was a verbal task (word generation task).

## Materials and methods

### Subjects

The study was approved by the ethics committee at the University of Alberta and all subjects gave full informed consent. Bipolar patients aged 18 – 65 who were right handed were eligible and were recruited from the Bipolar Clinic at the University of Alberta and by referral from colleagues. A full personal and family history was obtained and diagnosis was made using Diagnostic and Statistical Manual of Mental Disorders version IV (DSM-IV) criteria for bipolar disorder [11], using a the structured clinical interview for DSM-IV (SCID). All patients also had a physical examination including weight and blood pressure, biochemical screening including full blood count, urea and electrolytes, and measurement of liver and renal functioning. Patients who had any another Axis I diagnosis (including an anxiety disorder or drug or alcohol dependence or abuse), or who had clinically significant medical findings, were excluded from the study. Depressed symptomatology was determined by clinical interview using DSM-IV criteria. Symptom severity for depression was determined using the 17-item Hamilton Depression rating scale (HAM-D) [12], and the presence of manic symptoms was measured using the Young Mania Rating Scale (YMRS) [13]. Patients were determined as euthymic if they had been clinically stable for at least 3 months and had a HAM-D score of less than 12 and a YMRS of less than 6.

### Cognitive Tasks and Study Design

#### Working Memory Task

The working memory task consisted of two conditions. In the first condition there was a series of ten arrows pointing to the left or to the right. Subjects responded by pressing the left or right button. In the second condition a five-digit number was displayed for four seconds with the instruction 'Remember this Number'. Immediately after, a series of ten random single digits was presented. Subjects were asked to respond by pressing one button for yes and one for no if the single digit was or was not in the memorized number. In all, each condition was presented seven times in twenty-four second blocks pseudo-randomly arranged [14].

#### Word Generation Task

The word generation task had two conditions. In the first condition subjects were asked to repeat the word 'REST' silently until another instruction was given. In the second condition a series of ten single letters randomly chosen from the alphabet were displayed at 4-second intervals. During the display of a single letter, subjects responded by thinking of as many words as possible that begin with that letter and repeating them silently until another letter is presented or the 'REST' instruction appears. The experiment consisted of five forty second blocks of each condition beginning with 'REST' [14].

### Functional magnetic resonance imaging (fMRI) study design

All imaging parameters and tasks were identical to those described previously [14]. The fMRI images were collected on a 1.5T Siemens Sonata scanner, using a single shot EPI gradient echo sequence (TR = 4010 ms, TE = 50 ms, 1.7 × 1.7 mm, 4 mm thick) to acquire 30 contiguous slices obtained at an oblique angle along the AC-PC line. A high resolution T1 weighted MPRAGE sequence was also obtained for overlay of the functional analysis.

#### fMRI Data Analysis

Pre-processing and analysis was performed using Statistical Parametric Mapping (SPM), 1999 version [15]. All functional images were realigned during pre-processing to accommodate and correct for any head motion. Realignment was performed using a 6-parameter rigid body transformation and a mean image was created of the entire time series for each data set. Sessions with realignment parameters of greater than 4 mm in the direction of translation (along the x, y, z axis) were excluded from the final statistical analysis, as were sessions with motion greater than 0.05 radians in a rotational plane (pitch, roll, yaw). The mean image was then spatially normalized to the MNI template brain using a 12-parameter affine transformation with 12 non-linear iterations and 7 × 8 × 7 basis functions. The spatial transformations derived from normalizing the mean image to the template were then applied to the T2* weighted EPI functional images. After normalization, all volumes were resampled to 2 × 2 × 2 mm voxels using tri-linear interpolation in space. Finally, all functional images were smoothed with an 8-mm full width at half-maximum isotropic Gaussian kernel to compensate for between subject variability and allow Gaussian random field theory to give corrected statistical inferences [16]. Initial analysis was performed separately for each subject for each task. The model specified for each task was kept identical for all subjects and sessions to create identical design matrices. As part of this analysis three more pre-processing steps were performed using SPM99. First the data was high passed filtered to remove low-frequency drifts in the signal. In addition, the data was low pass filtered using the hemodynamic response function to remove high frequency noise. Effects due to global intensity fluctuations were removed when the data was proportionally scaled to a global mean of 100. The time series for each data set was analyzed according to the general linear model. Previously, we have performed a group analysis on 18 volunteers [14] by constructing a fixed effects model for each task. Regions of interest for each task were then compiled using the most significantly activated voxels from the group average generation maps and thus pre-determined regions of interested were examined. Region of interest (ROI) images were constructed using automated anatomical labeling software [17] running with MRIcro software [18]. In the present study we assessed activation in these previously identified ROIs.

For each subject the individual activation maps generated during single-subject analysis were used to identify the change in BOLD signal magnitude [14]. The BOLD signal intensity change was calculated based on regions of interest using the MARSBAR toolbox for SPM [19] over a seven-voxel sphere centered on the *most *significantly active voxel in each ROI. The fitted response (or BOLD signal intensity change) is expressed in percentage of whole brain mean. Because the global brain mean in the voxel-wise analysis was scaled to 100, this signal change represents the percentage of signal change with respect to the global mean intensity of the scaled images. The number of voxels over which the fitted response was calculated was kept small in order to minimize averaging over non-significant voxels or large veins [20]. Subsequently statistical calculations for BOLD signal magnitude were based on the average response calculated from the plateau portion of the hemodynamic response (eight seconds after stimulus origination until stimulus termination.

### Statistical analysis

The mean BOLD signal magnitude in each task was calculated over all the ROIs (for that task) and an analysis of variance (ANOVA) was used to detect between group differences at baseline and after lithium treatment. Means ± standard error of mean were compared between baseline and post-lithium scans using paired t-tests. The BOLD magnitude after treatment, for each patient, was then subtracted from the BOLD magnitude at baseline and the mean *change *in BOLD signal magnitude was calculated for each group (depressed and euthymic patients), in each task. A one-way analysis of variance (ANOVA) was carried out to determine between group differences in change in BOLD magnitude due to treatment. Post-hoc analyses using Student's *t*-tests were carried out for each ROI for each group, in each task to determine regional changes in BOLD signal following treatment. Analysis of possible age differences between groups was carried out using two-tailed Student's *t*-tests. Statistical significance levels of p < 0.05 were used throughout.

## Results

### Subjects

A total of 5 depressed bipolar patients and 5 euthymic bipolar patients took part in the study. Patient characteristics are shown in Table 1. The mean age of the euthymic patients was 30 ± 4.8 of whom 4 were female and of the depressed patients the mean age was 30 ± 2.1 of whom 4 were female. There was no statistically significant difference in age (t = 0.07, p = 0.94). Of the euthymic patients 4 were Bipolar Type II, compared to 3 of the depressed patients. Two of the euthymic patients were concurrently taking antidepressants, while 2 of the depressed patients were taking other psychiatric medication. There was no psychiatric comorbidity in this group. The mean HAM-D score of the euthymic group was 7.6 ± 1.4 at baseline and after lithium the mean score was 3.2 ± 1.3. The mean HAM-D score of the depressed group was 15.4 ± 0.7 at baseline and after lithium the mean score was 11.0 ± 2.6. This represents an approximate 30% decrease in HAM-D scores from the initial (mild) scores.

**Table 1:** Patient characteristics. This table shows the age, sex, type of bipolar disorder (BPD), either Type I (I) or Type II (II), as well as the score on the Hamilton Depression scale (HAM-D) and the Young mania rating scale (YMRS).

	Age	Sex	Type of BPD	Ham-D Baseline	Ham-D 14-days	YMRS Baseline	YMRS 14-Days	Medication at time of baseline scan
Euthymic Bipolar Patients								
1	26	F	II	11	1	7	5	Sertraline
2	22	F	II	8	3	4	5	
3	27	F	II	6	5	9	4	
4	26	F	II	10	7	7	4	Venlafaxine
5	49	M	I	3	0	3	2	
Depressed Bipolar Patients								
1	38	F	I	14	9	0	0	Quetiapine, venlafaxine, lamotrigine
2	27	F	II	15	2	7	4	
3	32	F	II	17	12	2	1	
4	28	M	I	14	17	1	1	Risperidone
5	27	F	II	17	15	6	4	

### Working memory task performance

There were no statistically significant differences between these groups on either measure of reaction time at baseline or after treatment (euthymic 529.66 ± 62.4 and depressed 485.91 ± 53.0, at baseline and euthymic 517.84 ± 36.5 and depressed 537.32 ± 23.8 after treatment for the control "arrow" condition; euthymic 907.85 ± 82.3 and depressed 885.50 ± 72.7, at baseline and euthymic 812.86 ± 25.3 and depressed 908.09 ± 67.5 after treatment, for the 5 digit task). There was also no significant difference between the groups on measures of the change in reaction time over after treatment. In the arrow task the change in reaction time was -11.52 ± 62.1 for the euthymic group and 51.4 ± 34.7 for the depressed group (p = 0.46). On the 5 digit task the mean change in reaction time for the euthymic group was -95.8 ± 88.3 and 22.6 ± 11.8 (p = 0.31).

### fMRI results

In the working memory task over all the regions of interest the mean BOLD signal ± SEM was 1.02 ± 0.04 for the euthymic group, and 1.12 ± 0.06 for the depressed group at baseline. After 14 days of lithium the mean signal ± SEM was 1.08 ± 0.05 for the euthymic group, and 1.14 ± 0.07 for the depressed group. Neither of these values varied statistically from baseline (p = 0.149 for the euthymic patients and p = 0.56 for the depressed patients). The mean change in BOLD signal due to treatment was 0.06 ± 0.04 for the euthymic group and 0.02 ± 0.04 for the depressed group. These mean changes in BOLD signal were not significantly different between the groups (F= 0.412, p = 0.522). The results for the BOLD signal change in each region of interest are shown in Table 2.

**Table 2:** Working Memory Task change in the BOLD signal for each region of interest pre-post lithium The change in each region of interest is shown for the working memory task (mean ± SEM). There are three treatment groups: healthy controls, euthymic bipolar patients, and depressed bipolar patients. Significant differences (and trends towards significant differences) between patient group and controls are shown as are significant differences (and trends towards significant differences) between euthymic bipolar patients and depressed bipolar patients.

Region of interest	Bipolar Euthymic Mean ± SEM	Bipolar Depressed Mean ± SEM
Cingulate gyrus	0.03 ± 0.12	-0.19 ± 0.12
Left dorsolateral prefrontal cortex	0.27 ± 0.14	0.03 ± 0.26
Right dorsolateral prefrontal cortex	-0.04 ± 0.05	-0.14 ± 0.22
Left inferior parietal gyrus	-0.15 ± 0.21	-0.10 ± 0.11
Right inferior parietal gyrus	-0.09 ± 0.18	-0.13 ± 0.17
Left precentral gyrus	-0.03 ± 0.29	0.10 ± 0.10
Right precentral gyrus	0.09 ± 0.14	-0.03 ± 0.05
Left superior parietal gyrus	-0.16 ± 0.23	-0.08 ± 0.23
Right superior parietal gyrus	-0.12 ± 0.23	-0.04 ± 0.11
Left inferior frontal gyrus	0.06 ± 0.31	0.02 ± 0.20
Right inferior frontal gyrus	0.10 ± 0.10	0.17 ± 0.15
Left insula	-0.03 ± 0.07	0.18 ± 0.15
Right insula	0.15 ± 0.11	0.08 ± 0.17
Left middle frontal gyrus	0.25 ± 0.17	0.36 ± 0.13
Right middle frontal gyrus	0.17 ± 0.31	-0.10 ± 0.20
Left superior frontal gyrus	0.04 ± 0.23	-0.28 ± 0.09
Right superior frontal gyrus	0.29 ± 0.27	0.03 ± 0.23
Left inferior occipital gyrus	0.31 ± 0.22	0.39 ± 0.26
Right inferior occipital gyrus	0.06 ± 0.18	0.19 ± 0.18

In the word generation task over all the regions of interest the mean BOLD signal ± SEM was 1.56 ± 0.10 for the euthymic group, and 1.49 ± 0.10 for the depressed group at baseline. After 14 days of lithium the mean signal ± SEM was 1.00 ± 0.07 for the euthymic group, and 1.53 ± 0.11 for the depressed group. The euthymic group, but not the depressed group, was statistically different from baseline (p = 0.000 for the euthymic patients and p = 0.61 for the depressed patients). The mean change in BOLD signal due to treatment was -0.57 ± 0.07 for the euthymic group and 0.04 ± 0.08 for the depressed group (F = 31.07, p = 0.000). Post-hoc ROI analysis showed that the mean changes in BOLD signal due to treatment were statistically significant in Broca's area, the left pre-central gyrus, and the supplemental motor area (Table 2).

## Discussion

The results from the present study show that following lithium there are differences between patients who are euthymic compared to those who are depressed. The results from the present study suggest that following lithium administration there is alteration in activation in specific brain regions during a word generation task in euthymic, but not depressed, bipolar patients. However, there appears to be no effect in either group in a working memory task. Interestingly, in previous work in healthy volunteers (unpublished information) using the same two cognitive tests we found that 14 days administration of lithium also led to a decrease in mean BOLD levels in the same regions of interest in the word generation task, but had no significant effects on the working memory task. It is possible that one of the reasons that the depressed group didn't show similar changes is that for these to be seen the patient must not be depressed. As we only tested these patients after 2 weeks of lithium, at which point they had only had a partial improvement in mood, it is possible that we would have seen changes in this group after a longer period.

Given findings from previous studies that in euthymic patients there are abnormalities of sustained attention, executive functioning, and verbal learning [1,2] it may have been anticipated that lithium may have also affected brain activation during the working memory task. Nonetheless, since lithium may itself have effects on verbal memory [4,5] it is conceivable that these two effects cancelled each other out. Also, one of the limitations of this study was the fact that a more detailed cognitive battery was not performed, and so it is not possible to determine what the cognitive abilities of this group of patients was before and after lithium treatment.

Other limitations of the study are the small size in each group. However, they are well matched on age, sex-distribution, and the number of type I and type II patients which are all factors that could potentially affect the results. Also, both groups each had 3 individuals in whom lithium was the only medication. Thus it is unlikely that these results reflect differences in any of these factors. Nonetheless, future studies should examine larger groups of patients, examine different mood stabilizers in addition to lithium, perhaps examine patients with more severe depression, and follow patients up for a longer period.

Recognizing the possible limitations of this study, one other fMRI study has examined euthymic and depressed bipolar patients [7], although this was on a single occasion. This study suggested there were both trait-related and state-related changes. Two other fMRI studies have also found differences between controls and euthymic patients [8,9]. There also appear to be differences between depressed bipolar patients and controls [21-23].

In conclusion, this is the first study to examine bipolar patients with fMRI before and after lithium administration. It suggests that lithium has effects on brain activation which are task, region, and state-dependent. These findings are in keeping with the current literature, which is clearly somewhat limited at present. More studies are required to answer some of the important outstanding questions.

**Table 3:** Word Generation Task change in the BOLD signal for each region of interest pre-post lithium The change in each region of interest is shown for the working memory task (mean ± SEM). There are three treatment groups: healthy controls, euthymic bipolar patients, and depressed bipolar patients. Significant differences (and trends towards significant differences) between patient group and controls are shown as are significant differences (and trends towards significant differences) between euthymic bipolar patients and depressed bipolar patients.

Region of interest	Bipolar Euthymic Mean ± SEM	Bipolar Depressed Mean ± SEM
Cingulate gyrus	-0.62 ± 0.22	-0.13 ± 0.22
Left dorsolateral prefrontal cortex	-0.47 ± 0.27	0.01 ± 0.23
Right dorsolateral prefrontal cortex	-0.76 ± 0.40	-0.14 ± 0.07
Left inferior parietal gyrus	-0.65 ± 0.39	-0.24 ± 0.33
Right inferior parietal gyrus	-0.60 ± 0.32	0.13 ± 0.14
Left precentral gyrus	-0.75 ± 0.27	0.68 ± 0.39 *^1^
Right precentral gyrus	-0.25 ± 0.16	-0.02 ± 0.03
Left superior parietal gyrus	-0.87 ± 0.30	-0.05 ± 0.47
Right superior parietal gyrus	-0.65 ± 0.21	-0.38 ± 0.66
Broca's area	-0.46 ± 0.25	0.89 ± 0.31 *^2^
Left superior temporal gyrus	-0.94 ± 0.26	-0.20 ± 0.22
Right superior temporal gyrus	-0.18 ± 0.05	-0.04 ± 0.07
Supplementary motor area	-0.77 ± 0.21	0.11 ± 0.12 *^3^
Thalamus	0.04 ± 0.14	-0.04 ± 0.15

*^1 ^p = 0.024 Euthymic vs Depressed

*^2 ^p = 0.015 Euthymic vs Depressed

*^3 ^p = 0.010 Euthymic vs Depressed
